# Titanium Niobium Oxide Ti_2_Nb_10_O_29_/Carbon Hybrid Electrodes Derived by Mechanochemically Synthesized Carbide for High‐Performance Lithium‐Ion Batteries

**DOI:** 10.1002/cssc.202002229

**Published:** 2020-11-17

**Authors:** Öznil Budak, Pattarachai Srimuk, Mesut Aslan, Hwirim Shim, Lars Borchardt, Volker Presser

**Affiliations:** ^1^ INM – Leibniz Institute for New Materials 66123 Saarbrücken Germany; ^2^ Department of Materials Science and Engineering Saarland University 66123 Saarbrücken Germany; ^3^ Inorganic Chemistry I Ruhr-University Bochum 44780 Bochum Germany

**Keywords:** batteries, lithium, hybrid material, mechanochemistry, titanium niobium oxide

## Abstract

This work introduces the facile and scalable two‐step synthesis of Ti_2_Nb_10_O_29_ (TNO)/carbon hybrid material as a promising anode for lithium‐ion batteries (LIBs). The first step consisted of a mechanically induced self‐sustaining reaction via ball‐milling at room temperature to produce titanium niobium carbide with a Ti and Nb stoichiometric ratio of 1 to 5. The second step involved the oxidation of as‐synthesized titanium niobium carbide to produce TNO. Synthetic air yielded fully oxidized TNO, while annealing in CO_2_ resulted in TNO/carbon hybrids. The electrochemical performance for the hybrid and non‐hybrid electrodes was surveyed in a narrow potential window (1.0–2.5 V vs. Li/Li^+^) and a large potential window (0.05–2.5 V vs. Li/Li^+^). The best hybrid material displayed a specific capacity of 350 mAh g^−1^ at a rate of 0.01 A g^−1^ (144 mAh g^−1^ at 1 A g^−1^) in the large potential window regime. The electrochemical performance of hybrid materials was superior compared to non‐hybrid materials for operation within the large potential window. Due to the advantage of carbon in hybrid material, the rate handling was faster than that of the non‐hybrid one. The hybrid materials displayed robust cycling stability and maintained ca. 70 % of their initial capacities after 500 cycles. In contrast, only ca. 26 % of the initial capacity was maintained after the first 40 cycles for non‐hybrid materials. We also applied our hybrid material as an anode in a full‐cell lithium‐ion battery by coupling it with commercial LiMn_2_O_4_.

## Graphical abstract

**Nanoscale hybridization**: mechanochemical carbide synthesis and subsequent thermal annealing yields unique nanohybrids of titanium niobium oxide and carbon. Capitalizing on the enhanced charge transport afforded by carbon and the lithium‐ion intercalation of the metal oxide, the best hybrid material displayed a specific capacity of 350 mAh g^−1^ at a rate of 0.01 A g^−1^ (144 mAh g^−1^ at 1 A g^−1^) within a potential range of 0.05–2.5 V vs. Li/Li^+^.

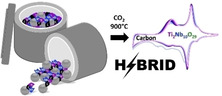

## Introduction

Electrochemical energy storage (EES) has become an integral part of the large‐scale implementation of renewable energy sources into the power grid, mobile computing/communication, and the transition of our fleet of internal‐combustion‐engine cars towards electric vehicles. One important EES technology, the lithium‐ion battery (LIB; featuring long cycle life, high energy density, and energy efficiency), capitalizes on the reversible charge storage intrinsic to lithiation/delithiation.^[1]^ Recent research focused on developing electrode materials with high charge‐storage capacity, cycling stable, and low production costs. Among different phases of titanium niobium oxide,^[2]^ Ti_2_Nb_10_O_29_ (TNO) draws considerable interest as an anode for LIBs.^[3]^ This is due to the theoretical capacity of TNO (396 mAh g^−1^) being comparable to or higher than that of commercially used lithium‐insertion materials such as graphite, TiO_2_, and Li_4_Ti_5_O_12_ (LTO).^[4]^ The enhanced energy‐storage capacity of TNO relates to its multiple redox couples of Ti^4+/3+^, Nb^5+/4+^, and Nb^4+/3+^.^[5]^

TNO suffers from a low electronic conductivity of about 5 ⋅ 10^−9^ S cm^−1^ and a poor rate capability.[^5c^, ^6^] To overcome this issue, Takashima et al. demonstrated an enhanced electronic conductivity of TNO with oxygen deficiencies (8 ⋅ 10^−6^ S cm^−1^) when reducing Ti^4+^ to Ti^3+^.^[6]^ Alternatively to modifying the electronic band structure of TNO, one can also employ conductive carbon in TNO electrodes.[^5e^, ^7^] Combining neat metal oxides with carbon may either be accomplished by mechanical mixing (composite electrodes) or by nanoscopic blending of the two components (hybrid materials) in the electrode.^[7b]^ The resulting electrochemical properties strongly depend on the synthesis approach and resulting carbon distribution. For example, TNO/reduced graphene oxide composites showed a specific capacity of 100 mAh g^−1^ at 1 A g^−1^,^[8]^ whereas electrospun TNO/carbon hybrid fiber materials showed a specific capacity of 140 mAh g^−1^ at a high specific current of 5 A g^−1^.^[7a]^ TNO/carbon composite electrodes prepared with 20 wt% acetylene black as a conductive additive delivered a specific capacity of 145 mAh g^−1^ at 4 A g^−1^ (30 °C),^[5b]^ while hybrid nanosized TNO/carbon onion electrodes delivered a specific capacity of 170 mAh g^−1^ at 5 A g^−1^.^[5e]^ Recently, Luo et al. reported that TNO/holey reduced graphene oxide can provide 175 mAh g^−1^ at 7 A g^−1^ (40 °C) while neat TNO can only deliver 120 mAh g^−1^.^[9]^ Carbon nanofiber/TNO displays fast charge‐transfer kinetics with a specific capacity of ca. 200 mAh g^−1^ at 8 A g^−1^ (60 °C),^[10]^ while N‐doped TNO/C core‐branch arrays exhibit a specific capacity of about 150 mAh g^−1^ at a high scan rate of 15 A g^−1^ (100 °C).^[11]^ In addition to those, TNO microspheres coated with N‐doped carbon shows a specific capacity of 200 mAh g^−1^ at 8 A g (40 °C), which is 50 mAh g^−1^ higher compared to that of neat TNO.^[12]^ Clearly, TNO hybrid materials can utilize the redox activity of the material better and deliver a faster charge/discharge rate than non‐hybrid materials.^[2a]^

Previous works have only explored the complex wet‐chemical synthesis of nanosized TNO particles and carbon hybrid electrodes for lithium‐ion batteries (LIBs). Our work introduces a new two‐step synthesis for producing carbide‐derived, nanosized TNO and carbon hybrid materials. The process involves first a mechanically induced self‐sustaining reaction (MSR) to obtain titanium niobium carbide (TNC) and a thermal annealing step to convert the material to TNO. The incomplete oxidation of carbides allows for the controlled design of metal oxide/carbon hybrids[^7c^, ^13^] and the adjustment of crystal structure defects (oxygen vacancies).^[14]^ To the best of our knowledge, our present work is the first to demonstrate a mixed metal oxide derived from a mixed metal carbide for battery applications. As a feature of the carbide‐derived oxide synthesis, it is possible to adjust the titanium‐to‐niobium molar ratio of the carbide to a value of 1 : 5 so that the resulting mixed metal oxide phase would be Ti_2_Nb_10_O_29_. The resulting TNO and carbon hybrid electrodes were tested as an anode material for LIBs using two different potential windows, namely, a narrow (1.0–2.5 V vs. Li/Li^+^) and a wide window (0.05–2.5 V vs. Li/Li^+^).

## Experimental Section

### Material synthesis

#### Synthesis of (Ti,Nb)C

Titanium niobium carbide (TNC) was synthesized by MSR at room temperature using niobium powder (99.9 %,<65 μm, chemPUR), titanium powder (325 mesh, abcr), and carbon. Two different types of carbon sources were used for the synthesis of TNC to evaluate their impact on the electrochemical performance. One of the carbons was commercially available carbon black type Super C45 (Timcal Graphite & Carbon), named as CB in this study. We also used carbon onions (abbreviated as OLC in this study),^[15]^ which were synthesized by annealing high‐purity detonation nanodiamond powder (NaBond) at 1300 °C under vacuum.^[16]^ The titanium powder, niobium powder, and carbon were first mixed with at a Ti/Nb/C molar ratio of ca. 1 : 5 : 5^[17]^ by using a Turbula shaker mixer for 15 min to acquire a homogenous powder mixture. Ten hard metal balls (96 wt% WC, 4 wt% Co) with a diameter of 10 mm and a mass of 7.6 g per ball were mixed with the obtained solid mixture into a 125 mL hard metal vial. Then, the vial was filled with Ar gas (H_2_O, O_2_<1 ppm) for 30 min. We kept the ball‐to‐powder mass ratio at 15. The vial was placed in one of the holders of the ball‐milling machine (Retsch PM400), and the same mass of the vial was also put on the opposite side of the vial holders to balance the system. The ball milling was carried out at a spinning rate of 300 rpm for 6 h by pausing every 15 min to avoid an escalating heat build‐up. The synthesized TNC samples were named based on the carbon sources. For instance, TNC‐OLC was synthesized by using carbon onions, whereas TNC‐CB was produced using carbon black.

#### Synthesis of Ti_2_Nb_10_O_29_

TNC‐CB and TNC‐OLC were used as precursors to synthesize Ti_2_Nb_10_O_29_ (TNO) by using the gas‐solid reaction. A quartz crucible with TNC powder was placed in the isothermal zone of the tube furnace (VG Scienta GP‐CVD), then the furnace was flushed with Ar gas at a flow rate of 100 cm^3^ min^−1^ for 2 h to ensure that an inert gas atmosphere was generated in the furnace before starting the thermal treatment. After that, the furnace was heated to 900 °C at a heating rate of 5 °C min^−1^ with an Ar flow rate of 50 cm^3^ min^−1^. The Ar atmosphere was chosen to avoid carbon burning during the heating. To obtain TNO‐CB‐CO_2_ and TNO‐OLC‐CO_2_, CO_2_ gas was also fed to the furnace with a flow rate of 50 cm^3^ min^−1^ right after reaching 900 °C; the furnace was then kept at 900 °C for 1 h.

We also studied TNO produced by thermal annealing in synthetic air instead of CO_2_. To produce TNO‐CB‐Air, and TNO‐OLC‐Air, only synthetic air was flushed at a flow rate of 50 cm^3^ min^−1^ during the holding temperature of 900 °C for 1 h. Afterwards, the sample was cooled to room temperature naturally using only Ar gas at a flow rate of 50 cm^3^ min^−1^.

### Material characterization

Scanning electron microscopy (SEM) images were obtained with a JEOL JSM 7500F field emission scanning electron microscope at an acceleration voltage of 3 kV. The samples were fixed on a stainless‐steel sample holder by using sticky carbon tape. The chemical compositions of the samples were quantified by energy‐dispersive X‐ray (EDX) spectroscopy with an X‐MAX‐150 detector (Oxford Instruments) attached to the SEM chamber. The samples were placed on a copper tape in the case of EDX analysis. The spectra of thirty spots were measured with an acceleration voltage of 15 kV and averaged.

The carbon content was quantified by chemical analysis using a MICRO Cube (Elementar Analysensysteme GmbH). The latter system was heated to reach a temperature of +1150 °C at the combustion tube and +850 °C at the reduction tube.

X‐ray powder diffraction (XRD) measurements were conducted with a D8 Discover diffractometer (Bruker AXS) with Cu_Kα_ radiation (wavelength: 0.15406 nm; voltage: 40 kV; current: 40 mA), a Goebel mirror in point focus (1 mm), and a VANTEC‐500 2D detector. The patterns were recorded at the positioned 2D detector from 17° to 97°2*θ* with the increment of 20°2*θ*; the total XRD measurement time was 60 min. The Rietveld refinement for TNC‐CB and TNC‐OLC, and Le Bail fitting analysis of TNO‐CB‐CO_2_, TNO‐OLC‐CO_2_, TNO‐CB‐Air, and TNO‐OLC‐Air were carried out by using the Bruker AXS software TOPAS 6.

Raman spectra were recorded with a Renishaw inVia system equipped with an Nd‐YAG laser of 532 nm, an excitation power of 0.5 mW at the surface of the samples, and an objective lens with a numeric aperture of 0.75. Spectra of 10 points were recorded for each sample, with 30 s acquisition time for three accumulations in the range of 100–3200 cm^−1^. Peak analysis was made by starting with baseline corrections and assuming Voigt peak profiles for the D mode, D* mode, G mode, and D** mode.^[18]^

### Electrochemical characterization

Electrodes were prepared from a slurry of samples (TNO‐CB‐CO_2_, TNO‐OLC‐CO_2_, TNO‐CB‐Air, and TNO‐OLC‐Air), polyvinylidene fluoride (PVDF, Alfa Aesar) as a binder, and carbon black (Super C65, Imerys Graphite & Carbon) as a conductive additive with a composition of 80 : 10 : 10 wt %, respectively. After mixing the required amount of sample and carbon black in a mortar with isopropanol for 10 min, the mortar was placed in an oven at 60 °C for 1 h to evaporate the isopropanol from the mixture. The obtained mixture was added to the prefabricated solution of PVDF and *N*‐methyl‐2‐pyrrolidone (NMP, Sigma‐Aldrich). The viscosity of the slurry was adjusted by an excess amount of NMP, and the resulting slurry was stirred overnight. The obtained slurry was coated on Cu foil at a wet thickness of 200 μm by using a doctor blade. The coated electrodes were dried in an oven at 110 °C under vacuum conditions overnight. The dry electrode sheets were cold pressed by using a rolling machine (HR01, MTI). The resulting electrode sheets were cut into a circle shape with a diameter of 10 mm. The average mass loadings of TNO‐CB‐CO_2_ and TNO‐OLC‐CO_2_ were 1.5 and 1.7 mg cm^−2^, respectively; for TNO‐CB‐Air and TNO‐OLC‐Air the mass loadings were 1.8 and 1.9 mg cm^−2^, respectively.

Coin cells (2032‐type) were assembled in an Ar‐filled glove box (O_2_, H_2_O<1 ppm) using a lithium chip (diameter: 12 mm) as a counter and reference electrode, 1 M lithium hexafluorophosphate (LiPF_6_) in an ethylene carbonate (EC) and dimethyl carbonate (DMC) mixture in the volumetric ratio of 1 : 1‐EC/DMC (Sigma‐Aldrich) as an electrolyte, and two pieces of Celgard 2325 (diameter: 18 mm) as separator. This setup is referred to as half‐cell.

All half‐cell electrochemical measurements were performed for the hybrid and non‐hybrid materials using two different potential window ranges, namely, 1.0–2.5 and 0.05–2.5 V vs. Li/Li^+^ at a scan rate of 0.05 mV s^−1^. Galvanostatic charge/discharge with potential limitation (GCPL) measurements were performed in an Arbin Battery Cycler by using the specific current range from 0.01 to 10 A g^−1^ to examine the rate capability; 0.1 A g^−1^ was used to observe cycling stability. Cyclic voltammetry (CV) results were carried out with a VMP300 system from Bio‐Logic multichannel potentiostat at scan rates of 0.05–2 mV s^−1^. The specific capacities were calculated based on the active material mass of the electrodes (excluding the mass of polymer binder) for the potential window of 0.05–2.5 V vs. Li/Li^+^, while the specific capacity of the electrochemical results in the potential window of 1.0–2.5 V vs. Li/Li^+^ was obtained by excluding the mass of carbon and polymer binder amount from TNO‐CB‐CO_2_ and TNO‐OLC‐CO_2_ electrodes. This capacity calculation method was preferred to obtain more reliable values due to the possible capacity contributions of carbon at low voltages.

Electrochemical impedance spectroscopy (EIS) measurements were carried out by using a VMP300 Bio‐Logic multichannel potentiostat at an applied AC voltage amplitude of 10 mV in the frequency range of 200 kHz to 10 mHz after 1 h resting of the half‐cells.

To better understand the structural changes of the materials at different lithiation/delithiation states, half‐cells were assembled for each of the materials. The cells were charged/discharged at the specific current of 0.1 A g^−1^ for 10 cycles in the potential range of 0.05–2.5 V vs. Li/Li^+^. Afterwards, the cells were held at 1.35 and 0.05 V vs. Li/Li^+^ for lithiation and at 1.35 and 2.5 V vs. Li/Li^+^ for delithiation until reaching an equilibrium current. Then the tested cells were disassembled in an Ar‐filled glove box (O_2_, H_2_O<1 ppm), and the electrodes were gently cleaned using DMC. Lastly, post‐mortem XRD measurements were performed on the cycled electrodes.

LiMn_2_O_4_ (LMO,<0.5 μm,>99 %, Sigma‐Aldrich) was chosen as a cathode for the full‐cell testing. The electrode was prepared by using LMO, carbon black (Super C65, Imerys Graphite & Carbon), and PVDF at a ratio of 80 : 10 : 10 (w/w), respectively. The LMO slurry preparation was carried out as aforementioned for the electrode preparation of hybrid and non‐hybrid materials, apart that the slurry was coated on Al foil at a wet thickness of 400 μm. The average mass loading of the obtained LMO electrode was 11.3 mg cm^−2^ with a diameter of 12 mm. Before conducting full‐cell experiments, an as‐prepared LMO electrode was tested for rate capability in half‐cell configuration using a potential window of 3.0–4.5 V vs. Li/Li^+^ following the procedures mentioned before.

For further evaluating the performance of the hybrid material (TNO‐OLC‐CO_2_), we assembled full‐cells by using custom‐built poly‐ether ether ketone cells described in elsewhere.^[19]^ The TNO‐OLC‐CO_2_ electrode was employed as a negative electrode (anode), LMO electrode as a positive electrode (cathode), and metallic lithium chip as a reference electrode. We used two pieces of Whatman GF/F glass fiber (diameter: 13 mm) as a separator and 1 M LiPF_6_ in EC/DMC (1 : 1 v/v, Sigma‐Aldrich) as the electrolyte. The built full‐cell was cycled at C‐rates of 0.1 C, 0.2 C, 0.5 C, 1 C, 2 C, and 5 C in the potential range of 0.5–4.5 V vs. Li/Li^+^ to obtain the Ragone plot. The specific energies of the full cell were calculated by integrating the voltage profile over the discharge time as in Equation 1:

(1) $$E_{\mathrm{sp}}=\frac{I\int_{t_{0}}^{t}U\left(t\right)dt}{m}$$

where *I* is applied current, *U* is the time‐dependent cell voltage, *t* is the time, and *m* is the mass of both electrodes (TNO‐OLC‐CO_2_, and LMO), separator, and current collectors (total dead‐mass: 20.1 mg), excluding the mass of the polymer binder.

The specific power of the full cell was calculated by dividing the specific energy by discharging time. The entire mass of the negative and positive electrodes was used for the calculation of the specific capacities, specific energies, and specific powers. The obtained full‐cell results were labeled as “TNO‐OLC‐CO_2_//LMO”.

## Results and Discussion

### Materials characterization

**Figure 1:**
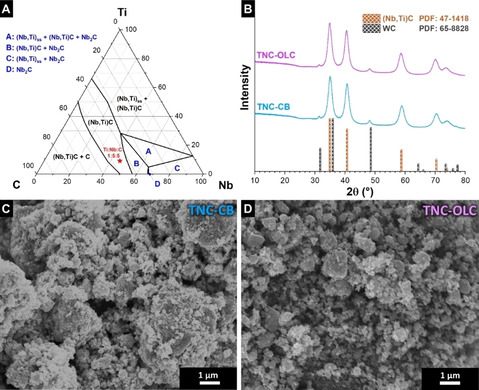
(A) Ternary phase diagram of Ti‐Nb‐C at 1600 °C (adapted from Ref. [17]). The red star marks the chemical composition used for the carbide synthesis in this work. “ss” stands for a solid solution. (B) XRD pattern of the titanium niobium carbide samples and the matched Bragg positions. SEM images of (C) TNC‐CB and (D) TNC‐OLC obtained after ball‐milling.

The formation of TNC occurs in the Ti‐Nb‐C system at 1600 °C (Figure 1A).[^17^, ^20^] To convert TNC to Ti_2_Nb_10_O_29_, we chose a Ti/Nb molar ratio of 1 : 5. Accordingly, the atomic carbon percentage was 45 % and those of Ti and Nb were 9.2 % and 45.8 %, respectively. The phase diagram shows an isothermal cut at 1600 °C and is not directly translatable to mechanochemical synthesis conditions. Our synthesis employs MSR, which offers a solvent‐free direct reaction route at ambient temperature and product uniformity.^[21]^ After the MSR of Ti, Nb, and C, we confirmed the formation of carbides by using X‐ray diffraction (Figure 1B). The diffraction pattern of TNC‐CB and TNC‐OLC were analyzed by using Rietveld refinement, assuming that the occupancy of Nb atoms in the carbide structure is five times higher than that of Ti atoms (Supporting Information, Table S1). We identified cubic ($Fm\bar{3}m$ ) titanium niobium carbide as a main phase and hexagonal ($P\bar{6}m2$ ) WC as an impurity phase (less than 2 wt% as determined by Rietveld analysis) related to the use of WC balls. The value of the *a*‐lattice parameter of 4.43 Å also confirms the presence of Ti within the (Ti,Nb)C lattice; for comparison, pure NbC would yield an *a*‐constant of 4.45 Å and pure TiC of 4.32 Å.^[17]^ The associated Ti/Nb ratio would be, based on the work of Ono and Moriyama, about 1 : 5.^[17]^ In comparison to TNC‐CB, TNC‐OLC has a similar lattice parameter and cell volume; however, the domain size of TNC‐CB (20 nm) is slightly larger than that of TNC‐OLC (15 nm). The SEM images of TNC‐CB and TNC‐OLC display irregular particles and agglomerates (Figure 1C and D). SEM images of CB and OLC are provided in the Supporting Information (Figure S1A and B).

For both TNC‐CB and TNC‐OLC, a Ti/Nb ratio of 1 : 5 was chosen to obtain pure Ti_2_Nb_10_O_29_ as the final product. The synthesis of TNO samples was carried out using two different atmospheric conditions: synthetic air and CO_2_. Synthetic air was used to produce the non‐hybrid materials (TNO‐CB‐Air, TNO‐OLC‐Air). This was accomplished by the complete volatilization of residual carbon from the carbide precursors and the full conversion of the carbide to oxide. The CO_2_ atmosphere allowed, via the Boudouard reaction, the preservation of some carbon from the carbide precursor, which has a dramatic influence on the electrochemical performance of the hybrid materials (TNO‐CB‐CO_2_, and TNO‐OLC‐CO_2_). To enable a better understanding of TNO formation by the CO_2_ oxidation method, thermogravimetric analysis coupled with mass spectroscopy (TGA‐MS) was conducted under the same synthesis conditions of the hybrid materials (Supporting Information, Figure S2). During heating under only Ar, CO was detected at around 800 °C for both hybrid material syntheses. Until reaching the synthesis holding temperature of 900 °C, the mass loss was higher for the TNO‐CB‐CO_2_ synthesis (3.6 %) than that of TNO‐OLC‐CO_2_ synthesis (1.2 %). While the heating process was performed under inert gas, the CO outgassing and the mass loss can only be explained by oxidation of free carbon caused by the possible surface functional groups of the free carbon. After the start of the holding temperature 900 °C under the gas mixture of Ar and CO_2_, the mass change showed an observable increase of CO outgassing, which is in good agreement with the Boudouard reaction [Eq. 2].

(2) $$\mathrm{CO}{}_{2}+C\vec{\leftarrow }2\;\mathrm{CO}$$

As a parallel reaction to the Boudouard reaction, the transformation of carbide to TNO might be described by Equation 3:

(3) $$2\;\mathrm{TiNb}{}_{5}C{}_{x}+15\;\mathrm{CO}{}_{2}\to \mathrm{Ti}{}_{2}\mathrm{Nb}{}_{10}O{}_{29}+\left(14+2\;x\right)\;C+\mathrm{CO}$$

As a result, the synthesis in a CO_2_‐containing atmosphere yielded a hybrid material (TNO‐CB‐CO_2_, TNO‐OLC‐CO_2_). The obtained hybrid and non‐hybrid materials display a Ti/Nb ratio of 1 : 5, with the hybrid materials TNO‐CB‐CO_2_ and TNO‐OLC‐CO_2_ having a carbon content of 1.2 wt% and 6.4 wt%, respectively (Table 1). The carbon‐content difference in the hybrid materials can be explained by the difference in morphology difference between the TNC‐CB and TNC‐OLC precursors. The smaller grain size and less degree of agglomeration of TNC‐OLC compared to TNC‐CB might lead to different reaction kinetics for Equations (2) and (3), resulting in a higher amount of carbon for TNO‐OLC‐CO_2_. Hence, the electrochemical performance of TNO‐OLC‐CO_2_ could be better than that of TNO‐CB‐CO_2_, as will be discussed later.

**Table 1:** Elemental composition analysis by energy‐dispersive X‐ray spectroscopy attached to a scanning electron microscope (SEM‐EDX) and carbon content measured by elemental analysis.

Sample	SEM‐EDX			Elemental
	Ti [at %]	Nb [at %]	O [at %]	C [wt %]
TNO‐CB‐CO_2_	3.8±0.6	19.6±1.5	67.4±3.5	1.2±0.1
TNO‐OLC‐CO_2_	3.6±1.2	18.9±5.3	60.8±6.2	6.4±0.1
TNO‐CB‐Air	4.0±1.0	21.7±5.0	73.1±6.8	0.1±0.1
TNO‐OLC‐Air	3.7±0.6	19.2±2.9	76.6±3.5	0.1±0.1

**Figure 2:**
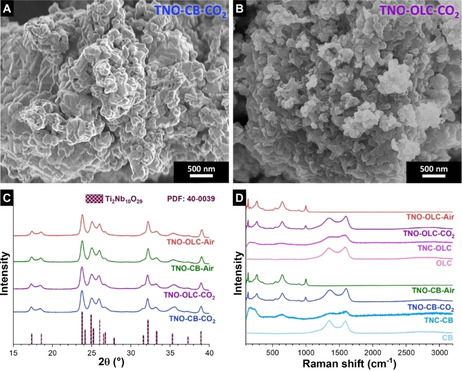
Material characterization of the TNO‐carbon hybrid materials, carbon onions (OLC) and carbon black (CB). SEM images of (A) TNO‐CB‐CO_2_ and (B) TNO‐OLC‐CO_2_. (C) X‐ray diffraction patterns of the TNO samples and the matched phase Bragg reflections. (D) Raman spectra of all samples.

The resulting nanomaterial after CO_2_ oxidation of titanium niobium carbide at 900 °C, TNO‐OLC‐CO_2_, exhibits an agglomerated morphology (Figure 2B). Also, non‐hybrid samples TNO‐CB‐Air and TNO‐OLC‐Air, and hybrid TNO‐CB‐CO_2_ sample show particles with an irregular agglomerated morphology (Figure 2A, and Supporting Information, Figure S3). The crystal structure characterization of TNO samples was further performed by Le Bail fitting of X‐ray diffractograms (Supporting Information, Table S2). The Le Bail fitting method was chosen since the obtained patterns exhibit only monoclinic (A12/*m*1) dititanium decaniobium oxide (Ti_2_Nb_10_O_29_, PDF 40‐0039; Wadsley‐Roth shear structure)^[22]^ with characteristic Bragg reflections at 23.8°, 25.0°, and 32.1 2*θ* (Figure 2C). The TNO‐CB‐CO_2_ and TNO‐CB‐Air samples show an average domain size of about 55 nm, whereas the domain size of TNO‐OLC‐CO_2_ and TNO‐OLC‐Air samples were slightly larger (63 and 88 m, respectively; Supporting Information, Table S2). This can be explained by possible different surface reactivity resulting from different domain sizes of the TNC‐CB and TNC‐OLC precursors.

The Raman spectra of TNO‐CB‐CO_2_, TNO‐OLC‐CO_2_, TNO‐CB‐Air, and TNO‐OLC‐Air are in alignment with the X‐ray diffraction results and confirm the presence of Ti_2_Nb_10_O_29_ (Figure 2D). The Raman bands located at 543, 648, 894, and 1000 cm^−1^ correspond to corner‐ and edge‐shared TiO_6_ octahedra and corner‐ and edge‐shared NbO_6_ octahedra, respectively.^[23]^ Apart from TNO‐CB‐Air and TNO‐OLC‐Air, all hybrid materials show the characteristic peaks of incomplete graphitic sp^2^‐hybridized carbon, namely, the D mode and the G mode at around 1358 and 1600 cm^−1^, respectively.^[24]^ As seen in Table 2, the full‐width at half‐maximum (FWHM) of the carbon‐related G mode and the *I*_D_/*I*_G_ ratio of TNO‐CB‐CO_2_ and TNO‐OLC‐CO_2_ are similar, namely, 73 and 70 cm^−1^ and 2.6 and 2.7, respectively. However, the FWHM of the D mode of TNO‐CB‐CO_2_ (154 cm^−1^) and TNO‐OLC‐CO_2_ (167 cm^−1^) are slightly different. Due to the fact that the D mode corresponds to the breathing mode of sp^2^‐hybridized carbon rings and is active in the presence of defects, TNO‐CB‐CO_2_ may exhibit a slightly higher degree of graphitic ordering compared to TNO‐OLC‐CO_2_. We suspect that the higher degree of graphitic carbon could lead to better rate capability of the materials, but the distribution of the carbon phase is also of vital importance for the electrochemical performance. Hence, we will discuss the influence of different carbon precursors based on electrochemical performance later.

**Table 2:** Raman spectra peak analysis of the carbon D and G modes.

Sample	Mode	Position [cm^−1^]	FWHM [cm^−1^]	*I*_D_/*I*_G_
CB	D mode	1351±2	128±6	2.5±0.3
	G mode	1598±1	76±2	
TNC‐CB	D mode	1365±4	210±9	2.4±0.5
	G mode	1604±3	101±6	
TNO‐CB‐CO_2_	D mode	1363±3	154±8	2.6±0.5
	G mode	1607±2	73±3	
OLC	D mode	1347±2	160±4	3.3±0.4
	G mode	1604±2	84±2	
TNC‐OLC	D mode	1374±7	199±9	1.3±0.2
	G mode	1583±9	130±9	
TNO‐OLC‐CO_2_	D mode	1347±6	167±4	2.7±0.5
	G mode	1604±1	70±4	

### Electrochemical analysis

The electrochemical performance of the as‐synthesized materials was tested by using two different operational potential windows: the typical potential range of 1.0–2.5 V vs. Li/Li^+^, which most previous works have explored, and additionally within a widened range of 0.05–2.5 V vs. Li/Li^+^. The latter was included to further study TNO as an anode material and to specifically address performance stability.

**Figure 3:**
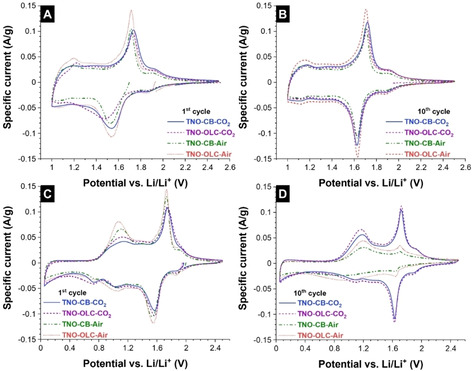
CVs at a scan rate of 0.05 mV s^−1^ of the first cycle (A, C) and the 10^th^ cycle (B, D) of the samples for the potential window of 1.0–2.5 V vs. Li/Li^+^ (A, B) and 0.05–2.5 V vs. Li/Li^+^ (C, D).

First, the non‐hybrid (TNO‐CB‐Air and TNO‐OLC‐Air) and hybrid materials (TNO‐CB‐CO_2_, and TNO‐OLC‐CO_2_) were anodically scanned from open‐circuit potential to 1.0 V vs. Li/Li^+^ using CV. Figure 3 shows that there are multiple redox couples during lithium intercalation into the TNO structure. The peak at 1.8–1.9 V vs. Li/Li^+^ indicates the redox couple of Ti^4+^/Ti^3+^, whereas the sharp peak at 1.4–1.6 V vs. Li/Li^+^ represents the Nb^5+^/Nb^4+^ transition. We also observed a broad peak at around 1.0–1.1 V vs. Li/Li^+^, suggesting another redox couple of Nb (Nb^4+^/Nb^3+^).^[25]^ When the electrode was cathodically scanned from 1.0 to 2.5 V vs. Li/Li^+^, we see three redox peaks that indicate the delithiation process. The 10^th^ CV cycle is shown in Figure 3B. For all four electrodes, the CV areas are almost identical for TNO‐CB‐CO_2_, TNO‐OLC‐CO_2_, TNO‐CB‐Air, and TNO‐OLC‐Air. This observation indicates that there is no significant change in the specific capacity. In good agreement with the galvanostatic charge/discharge results (Supporting Information, Figure S4A), TNO‐CB‐CO_2_, TNO‐CB‐Air, and TNO‐OLC‐Air exhibit a specific capacity of ca. 275 mAh g^−1^ at a specific current of 0.01 A g^−1^. Compared to the other materials, TNO‐OLC‐CO_2_ delivers a slightly lower capacity (250 mAh g^−1^). Considering a theoretical capacity of Ti_2_Nb_10_O_29_ is 396 mAh g^−1^ (22 Li^+^ per unit formula), our materials deliver only 70 % of the theoretical value within the potential of 1.0–2.5 V vs. Li/Li^+^. Possibly, the operational potential of 1.0–2.5 V vs. Li/Li^+^ does not allow the full insertion/de‐insertion of lithium. Thus, we also explored an extended potential regime to further quantify the maximum lithium storage capacity and life cycle of the TNO.

To understand the improved rate capability, a kinetics analysis (*b*‐value) was applied by using the peak current obtained from CVs at different scan rates (Supporting Information, Figure S5). The data was fitted using Equation 4:

(4) $$i=a\vartheta^{b}$$

where *a* and *b* are fitting variables. For a *b*‐value of 0.5, a process would be limited by diffusion, whereas a *b*‐value of 1 is typical for a surface‐controlled mechanism.^[26]^ At the potential range of 1.0–2.5 V vs. Li/Li^+^, the analyzed data indicate that the lithiation/delithiation process is diffusion controlled (Supporting Information, Figure S5).

The cycling stability was also tested at 0.1 A g^−1^, and the results are shown in the Supporting Information, Figure S4B. The non‐hybrid materials, TNO‐CB‐Air and TNO‐OLC‐Air, have an initial capacity of 203 and 238 mAh g^−1^, while the initial capacity values are 217 and 225 mAh g^−1^ for TNO‐CB‐CO_2_ and TNO‐OLC‐CO_2_ hybrid materials, respectively. Compared to hybrids, the performance decays faster for the non‐hybrid materials during the first 50–100 charge/discharge cycles, remaining at a rather constant performance level thereafter. For example, the capacity loss after 150 cycles of hybrid TNO‐CB‐CO_2_ is only 7.2 % whereas the loss in initial capacity for the non‐hybrid TNO‐CB‐Air is more than two times as high (16.0 %). Specifically, TNO‐CB‐Air and TNO‐OLC‐Air have a 29.2 % and 29.4 % capacity decrease after 500 cycles, while TNO‐CB‐CO_2_ and TNO‐OLC‐CO_2_ only lose 20.6 % and 24.9 %, respectively, of their initial capacities after 500 cycles. The better cycling performance of the hybrid material may result from the better charge percolation/conductivity via carbon in the hybrid materials; carbon might also protect the active material from deterioration of the electrochemical properties.[^5d^, ^27^] The rate capability results within the narrow operating potential range did not indicate universally superior charge percolation of the hybrid materials; therefore, the lower cycling stability of non‐hybrid TNO may be caused by the dynamic volume change during the charge/discharge process.^[5a]^

CVs of carbon‐hybrid TNO materials (TNO‐CB‐CO_2_, TNO‐OLC‐CO_2_) performed within the wider potential window of 0.05–2.5 V vs. Li/Li^+^ (Figure 3C and D) show a different behavior compared to the non‐hybrid materials (TNO‐CB‐Air, TNO‐OLC‐Air). In the first cycle, three reduction peaks between 0.8 and 2.1 V vs. Li/Li^+^ indicate the transitions of Ti^4+^/Ti^3+^, Nb^5+^/Nb^4+^, and Nb^4+^/Nb^3+^ (Figure 3C). When scanning to lower potentials, we observed a peak at 0.7 V vs. Li/Li^+^, which could be related to the SEI (solid‐electrolyte interface) formation. During the cathodic scan, the oxidation peak at 1.1 V vs. Li/Li^+^ is much more pronounced compared to the potential range 1.0–2.5 V vs. Li/Li^+^. This means that when applying a potential lower than 1.0 V vs. Li/Li^+^, it might trigger full lithium intercalation into the TNO structure, especially for the redox couple of Nb^4+^/Nb^3+^. Subsequently, the electrochemical behavior completely changes in the 10^th^ cycle (Figure 3D). Hybrid materials (TNO‐CB‐CO_2_ and TNO‐OLC‐CO_2_) can maintain the peak at 1.9 V vs. Li/Li^+^ (Ti^4+^/Ti^3+^), 1.6 V vs. Li/Li^+^ (Nb^5+^/Nb^4+^), and 1.1 V vs. Li/Li^+^ (Nb^4+^/Nb^3+^) while the non‐hybrid materials (TNO‐CB‐Air, and TNO‐OLC‐Air) lose the activity of Ti^4+^/Ti^3+^ and Nb^5+^/Nb^4+^.

**Figure 4:**
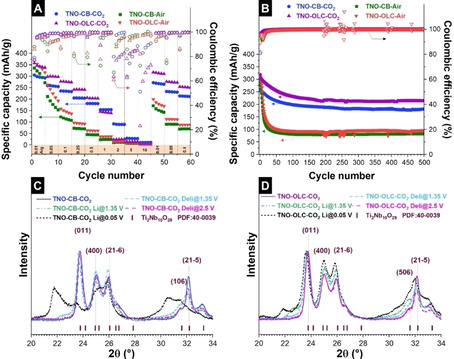
(A) Rate capability of the hybrid and non‐hybrid materials in the potential window of 0.05–2.5 V vs. Li/Li^+^. (B) Cycling stability of the hybrid and non‐hybrid materials in the potential window of 0.05–2.5 V vs. Li/Li^+^ at a specific current of 0.1 A g^−1>^. XRD patterns of (C) TNO‐CB‐CO_2_ and (D) TNO‐OLC‐CO_2_ electrodes at different lithiated (“Li”: values at different potentials vs. Li/Li^+^) and delithiated states (“Deli”: values at different potentials vs. Li/Li^+^), respectively.

The galvanostatic charge/discharge cycling was carried out for a potential range of 0.05–2.5 V vs. Li/Li^+^, as shown in Figure 4A. Except for TNO‐CB‐CO_2_, all samples exhibit an initial capacity of about 350 mAh g^−1^. The latter value corresponds to 88 % of the theoretical capacity of TNO. These high values are, to the best of our knowledge, the highest ones obtained for Ti_2_Nb_10_O_29_/carbon materials (either composite or hybrid), as shown in Supporting Information, Table S3. The only higher value was reported for another type of TNO, namely TiNb_2_O_7_/C nanoporous microspheres (393 mAh g^−1^ at 0.1 A g^−1^).^[28]^

Some differences can be seen between the hybrid and non‐hybrid materials for continuous cycling. Specifically, the non‐hybrid materials (TNO‐CB‐Air and TNO‐OLC‐Air) show a more rapid loss of capacity, while the performance of hybrid samples is more stable within each specific current level. The hybrid materials (TNO‐CB‐CO_2_, TNO‐OLC‐CO_2_) can still provide ca. 150 mAh g^−1^ at a high specific current of 1 A g^−1^. This might be explained by the delithiation at 1.1 and 1.7 V vs. Li/Li^+^ changing to more surface‐controlled reactions for the hybrid materials (*b*‐value: 0.7) in comparison to the non‐hybrid materials (*b*‐value: 0.6)as revealed by kinetics analysis (Supporting Information, Figure S6). In addition, the amount of conductive carbon in the electrodes and a uniform carbon distribution to generate an electron conduction path will affect the rate handling ability.^[29]^ Therefore, the better rate capability of TNO‐OLC‐CO_2_ compared to TNO‐CB‐CO_2_ might be correlated with the higher amount of carbon and better carbon distribution (Table 1, and Supporting Information, Figure S7).

To further understand the better rate capability of hybrid materials than that of non‐hybrid materials, EIS of the half‐cells was performed, and the corresponding Nyquist plot is shown in the Supporting Information, Figure S8, with the equivalent circuit being shown as an inset (R_1_+(Q/(R_2_+W))+C). The electrical impedance spectra are composed of one semicircle and one linear regime. These features are indicative of a charge‐transfer element and a mass‐transport element. In the equivalent circuit, R_1_, R_2_, Q, W, and C stand for electrolyte and cell component resistance, charge‐transfer resistance, constant phase element, the Warburg impedance, and constant phase element based on an ideal capacitor, respectively. The fitted charge‐transfer resistance values of the hybrids TNO‐CB‐CO_2_ and TNO‐OLC‐CO_2_ are 168.8 Ω and 138.7 Ω, respectively, which are significantly lower than the charge‐transfer resistance of the non‐hybrid materials TNO‐CB‐Air and TNO‐OLC‐Air (Supporting Information, Table S4). The lower charge‐transfer resistance indicates faster charge‐transfer kinetics in the hybrid materials due to improved electronic conductivity. Also, the higher carbon content in TNO‐OLC‐CO_2_ compared to TNO‐CB‐CO_2_ may align with both a lower charge‐transfer resistance and an improved rate capability.

When returning to a low rate (0.01 A g^−1^) after 45 cycles, hybrid materials can largely recover their initial capacities while non‐hybrid materials can only provide ca. 44 % of their initial capacities. The cycling stability of the samples within the potential window of 0.05–2.5 V vs. Li/Li^+^ is depicted in Figure 4B. In this enlarged potential window, the cycling stabilities of the hybrid and non‐hybrid materials show a significant difference. The initial capacities of the hybrid material (TNO‐OLC‐CO_2_) and non‐hybrid materials (TNO‐CB‐Air and TNO‐OLC‐Air) are very similar at a level of about 300 mAh g^−1^. In contrast, the hybrid electrode (TNO‐CB‐CO_2_) exhibits a specific capacity of ca. 250 mAh g^−1^. The hybrid materials, TNO‐CB‐CO_2_ and TNO‐OLC‐CO_2_, preserve 70 % and 67 %, respectively, of their initial capacities after 500 cycles. For comparison, the non‐hybrid materials retain only 27 % of their initial capacities after the first 50 cycles.

To further understand why the hybrid materials outperform their non‐hybrid counterparts, we conducted half‐cell experiments of all samples by holding them at certain voltages for different lithiation/delithiation states after the first 10 cycles for the potential window of 0.05–2.5 V vs. Li/Li^+^. After the cells reached an equilibrium current, the cells were disassembled; we used X‐ray diffraction to analyze the structural changes of TNO (Figure 4C and D). While the Bragg reflections (011) and (21‐5) in the lithiated state at 1.35 V vs. Li/Li^+^ of TNO‐CB‐CO_2_ shift to lower angles, differences become more visible after reaching the fully lithiated state at 0.05 V vs. Li/Li^+^ (Figure 4C). Further, the Bragg reflections of (011) and (21−5) proceed to shift towards larger *d* spacings (lower angles), and the new reflections at ca. 21.8°2*θ*, and 30.5°2*θ* appears when reaching the fully lithiated state. These data suggest that as TNO is fully lithiated, a new crystal phase emerges.^[5a]^ For TNO‐OLC‐CO_2_, the new phase reflections become visible even for the lithiation state of 1.35 V vs. Li/Li^+^ at 22.6° and 30.7°2*θ* (namely: TNO‐OLC‐CO_2_ Li@1.35 V), then they move to lower angles 21.8° and 30.5°2*θ* after reaching the full lithiation state at 0.05 V vs. Li/Li^+^ (Figure 4D). In both hybrid materials, two phases coexist after reaching the full lithiation state at 0.05 V vs. Li/Li^+^.^[5a]^ At the deepest lithiation state (0.05 V vs. Li/Li^+^), TNO‐CB‐CO_2_ exhibits broader and higher intensity reflections for the aforementioned new phase positions (ca. 21.8° and 30.5°2*θ*) than that of TNO‐OLC‐CO_2_. We suspect that the higher amount of carbon in the TNO‐OLC‐CO_2_ than that of TNO‐CB‐CO_2_ (Table 1) might suppress a volume change as well as an anisotropic lattice change. The reflections of the hybrid materials regain their initial Bragg positions after returning to the fully delithiated state at 2.5 V vs. Li/Li^+^ (Figure 4C and D). In contrast, the X‐ray diffractograms of the non‐hybrid materials at different lithiation and delithiation states show neither new reflections nor significant shifting of the reflections (Supporting Information, Figure S9).

A possible explanation for the observed electrochemical degradation may be the formation of microcracks as a result of unit‐cell expansion during deep lithiation at the potential of 0.05 V vs. Li/Li^+^.^[30]^ This process may be suppressed for the hybrid material because of the nanoscopic level blending of active material and conductive carbon.^[31]^ Although this explanation is in alignment with the literature and the observed electrochemical performance, further work is needed to verify this degradation mechanism for our materials.

**Figure 5:**
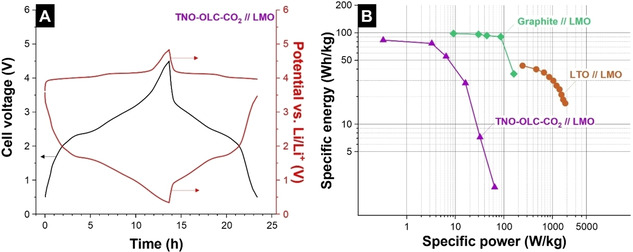
(A) Voltage profile of the full cell with TNO‐OLC‐CO_2_ as negative electrode and LMO as positive electrode at 0.1 C. The potentials are plotted as cell voltage and as the corresponding electrode potentials. (B) Ragone plot of the TNO‐OLC‐CO_2_//LMO full cell, graphite//LMO (adapted from Ref. [33]) and LTO//LMO (adapted from Ref. [34]).

To demonstrate our hybrid material as the anode in a full‐cell lithium‐ion battery, we paired our anodes with a commercially available LiMn_2_O_4_ (LMO) cathode (Figure 5). The charge/mass balance of the cell was based on the specific capacity of both anode and cathode. After careful analysis of the half‐cell data, the mass ratio between anode and cathode was 0.3 for the full‐cell measurement. We used a C rate of 0.1 C in a cell voltage range of 0.5–4.5 V (Figure 5A). The voltage profile of the positive electrode displays a lithiation/delithiation plateau at 4.0 V vs. Li/Li^+^, which is attributed to the Mn^3+^/Mn^4+^ redox couple.^[32]^ The voltage profile of TNO‐OLC‐CO_2_ exhibits three different slopes at around 1.9, 1.7, and 1.1 V vs. Li/Li^+^, which agree with the CVs shown in Figure 3C and D. The calculated energy efficiency from the voltage profile of the cell is 64 % at 0.1 C. The full‐cell results of TNO‐OLC‐CO_2_//LMO are compared with full‐cell results of graphite and LTO coupled with LMO in a Ragone plot (Figure 5B).

At a low C rate of 0.1 C, the TNO‐OLC‐CO_2_//LMO full‐cell delivers a maximum energy of 83 Wh kg^−1^, which is lower than the highest specific energy of graphite, whereas LTO can only provide 98 Wh kg^−1^, and 44 Wh kg^−1^, respectively. However, these preliminary results can be further optimized by eliminating the dead mass. In our results, without considering the package mass, only 42 % is the active mass; hence, by decreasing the mass of the current collector, the performance of this cell could increase significantly. As noted, the specific power of TNO‐OLC‐CO_2_//LMO (64.8 W kg^−1^) is lower than that of graphite and LTO (161 and 1.8 kW kg^−1^, respectively). This poorer power performance of the TNO‐OLC‐CO_2_//LMO full cell might be explained by the reduced rate capability of commercial LMO (Supporting Information, Figure S10).

## Conclusions

We present a facile two‐step synthesis method for carbide‐derived electrode materials for lithium‐ion battery (LIB) applications based on Ti_2_Nb_10_O_29_ (TNO) and carbon. The prepared TNO hybrid and non‐hybrid electrodes were tested in two different potential windows: a narrow range (1.0–2.5 V vs. Li/Li^+^) and a wide range (0.05–2.5 V vs. Li/Li^+^). While the performance of the hybrid and non‐hybrid electrodes for the narrow potential window show no significant differences, hybrid electrodes exhibit superior performance compared to the non‐hybrid electrodes for the wider potential window. X‐ray diffraction analysis of the electrodes held at certain lithiation/delithiation states after 10 cycles for the large potential window revealed that hybrid electrodes have a two‐phase region after reaching the fully lithiated state while non‐hybrid electrodes show no significant structural change. The initial capacities of all electrodes are similar (ca. 350 mAh g^−1^), but there is a faster electrochemical performance decay of non‐hybrid materials.

## Supporting Information

Results from Rietveld refinement analysis, Le Bail analysis, scanning electron micrographs, X‐ray diffractograms, illustrations of crystal structures, electrochemical results.

## Conflict of interest

The authors declare no conflict of interest.

## Supporting information

As a service to our authors and readers, this journal provides supporting information supplied by the authors. Such materials are peer reviewed and may be re‐organized for online delivery, but are not copy‐edited or typeset. Technical support issues arising from supporting information (other than missing files) should be addressed to the authors.

Supplementary
