# Supplementary material for: Titanium Niobium Oxide Ti2Nb10O29/Carbon Hybrid Electrodes Derived by Mechanochemically Synthesized Carbide for High‐Performance Lithium‐Ion Batteries
Source: ChemSusChem. 2020 Nov 17;14(1):398–407. doi: 10.1002/cssc.202002229 (PMC7839535; doi:10.1002/cssc.202002229)
Supplement: Supplementary file 1 — Supplementary [file CSSC-14-398-s001.pdf]

ChemSusChem

Supporting Information

# Titanium Niobium Oxide $Ti_2Nb_{10}O_{29}$/Carbon Hybrid Electrodes Derived by Mechanochemically Synthesized Carbide for High-Performance Lithium-Ion Batteries

Öznil Budak, Pattarachai Srimuk, Mesut Aslan, Hwirim Shim, Lars Borchardt, and Volker Presser*© 2020 The Authors. ChemSusChem published by Wiley-VCH GmbH. This is an open access article under the terms of the Creative Commons Attribution License, which permits use, distribution and reproduction in any medium, provided the original work is properly cited.

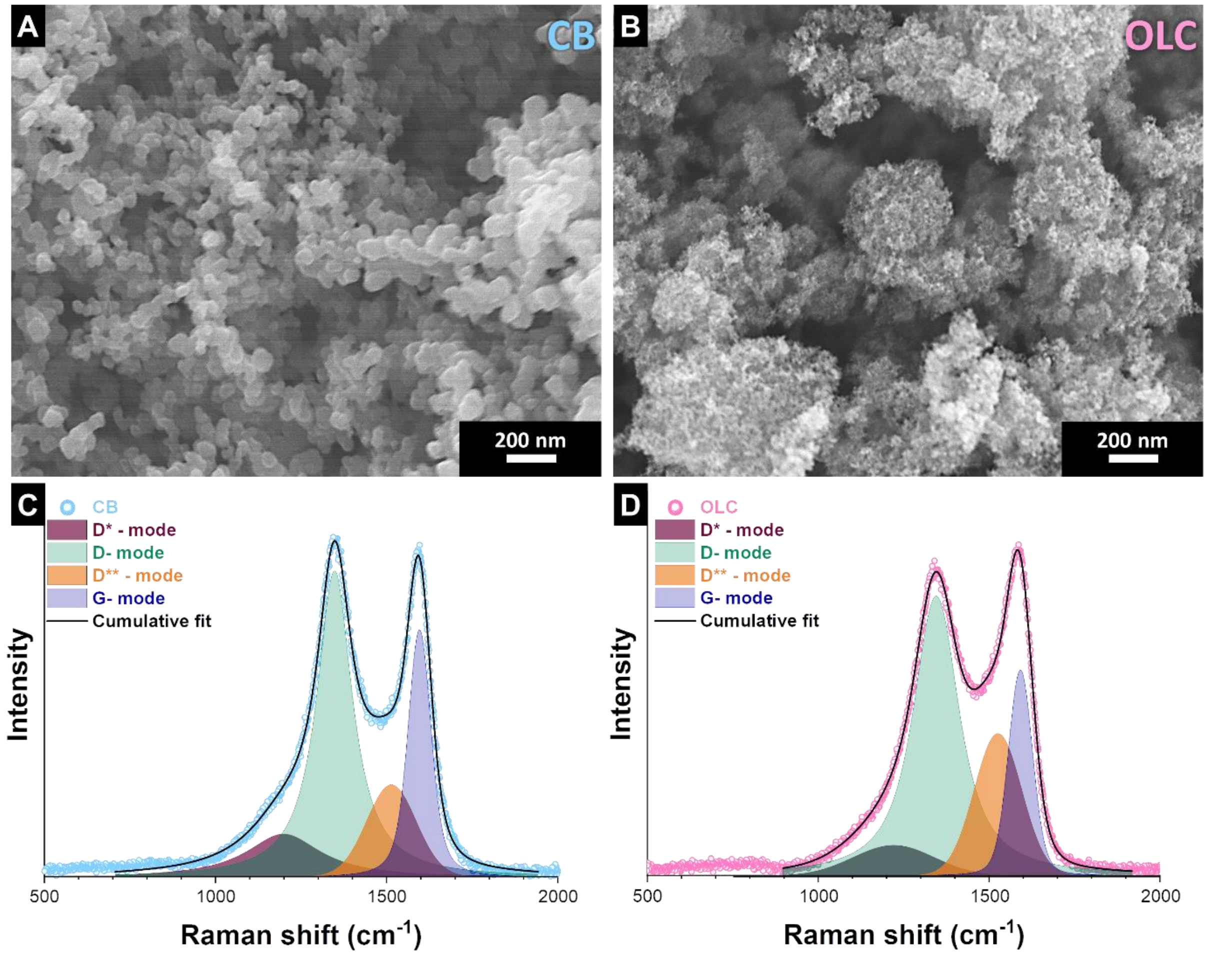


**Fig. S1.** (A-B) Scanning electron micrographs of (A) CB, and (B) OLC. Fitted Raman spectra of (C) CB, and (D) OLC.

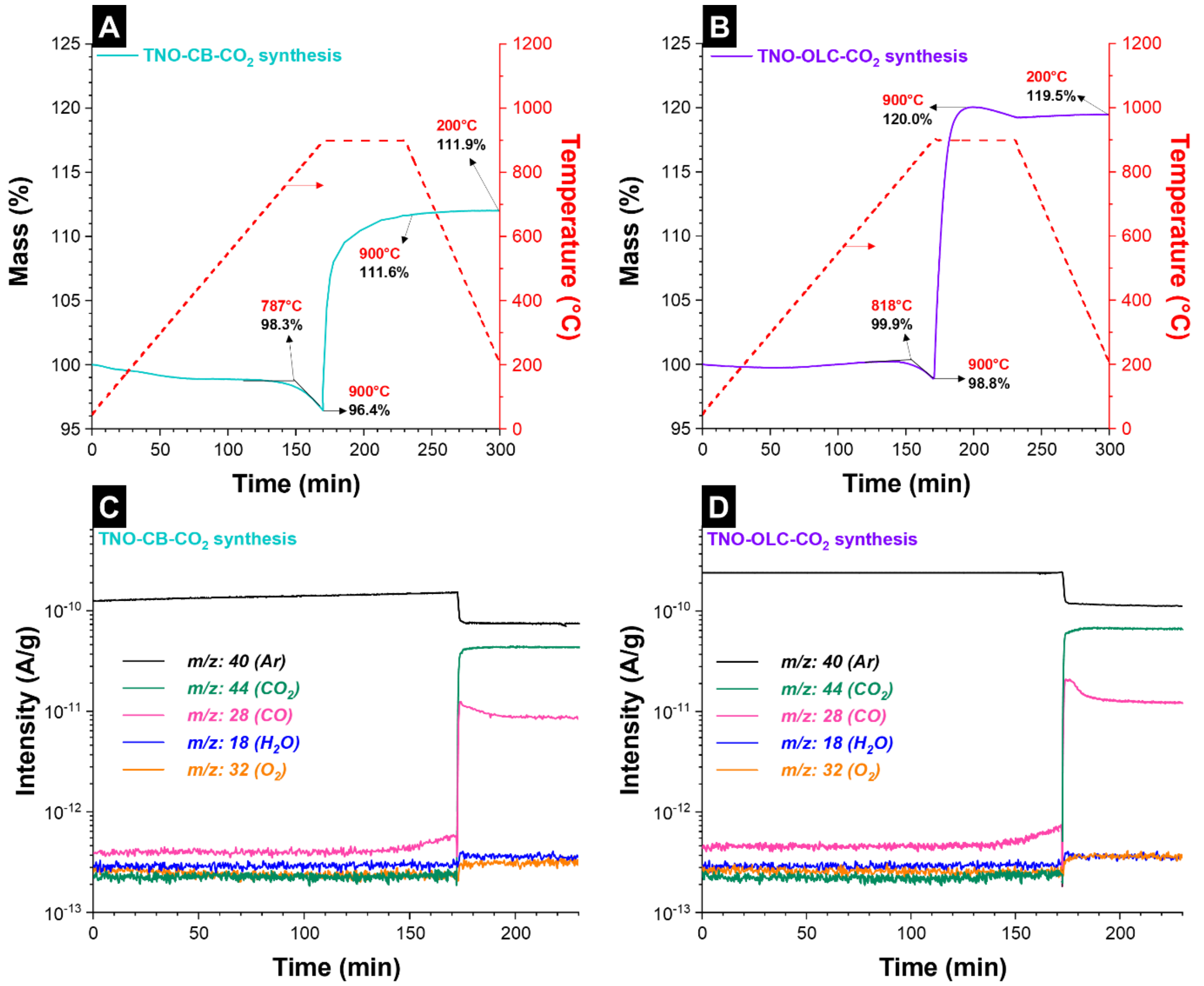


**Fig. S2.** (A-B) Thermograms of (A) TNO-CB-$CO_2$, and (B) TNO-OLC-$CO_2$ synthesis following the synthesis protocol of hybrid materials. The corresponding mass spectra of TGA-MS measurements of (C) TNO-CB-$CO_2$, and (D) TNO-OLC-$CO_2$ synthesis.

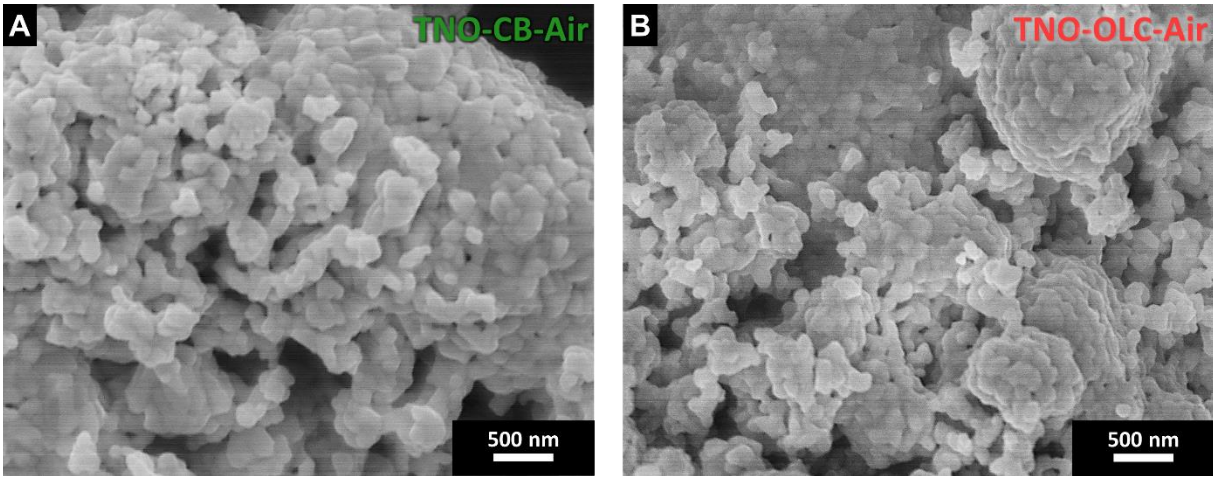


**Fig. S3.** (A-B) Scanning electron micrographs of (A) TNO-CB-Air, and (B) TNO-OLC-Air.

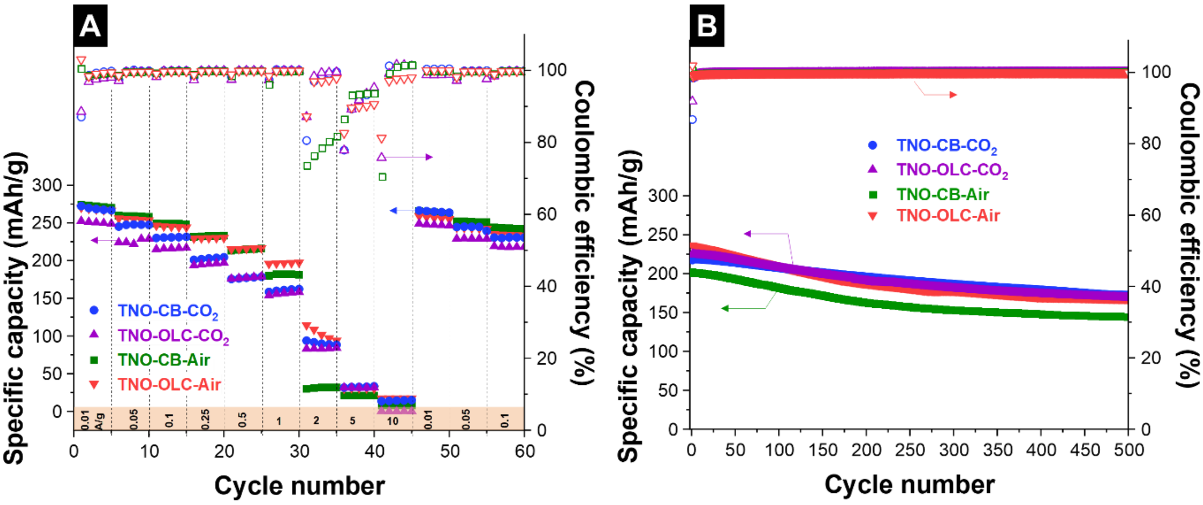

**Fig. S4.** (A) Rate handling performance of the hybrid and non-hybrid materials in the potential window of 1.0-2.5 V vs. Li/Li$^+$. (B) Cyclic stability performance of the hybrid and non-hybrid materials in the potential window of 1.0-2.5 V vs. Li/Li$^+$.

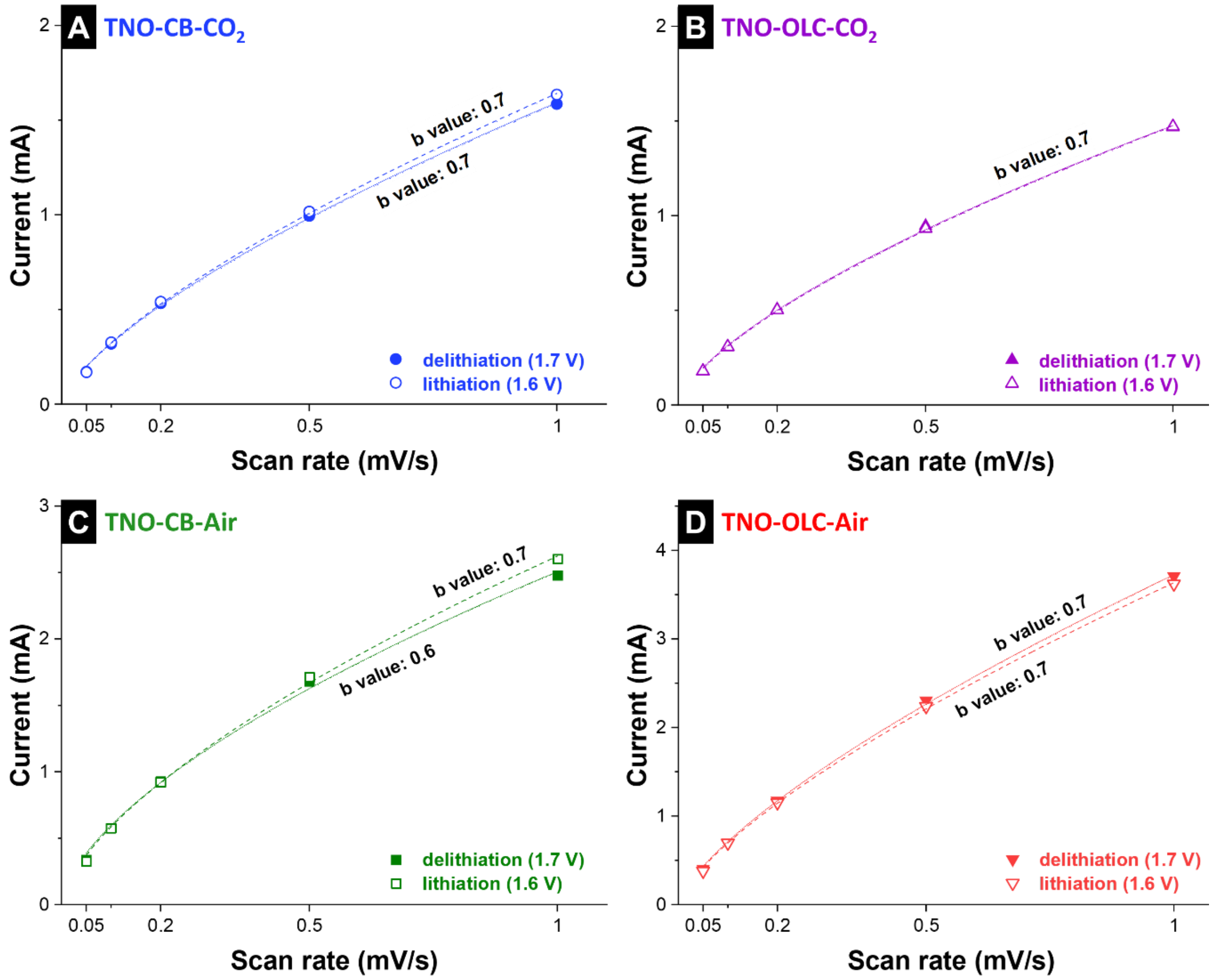

**Fig. S5.** Kinetic analysis of (A) TNO-CB-$CO_2$, (B) TNO-OLC-$CO_2$, (C) TNO-CB-Air, and (D) TNO-OLC-Air calculated from cyclic voltammograms at the different scan rates for the potential window of 1.0-2.5 V vs. Li/Li$^+$.

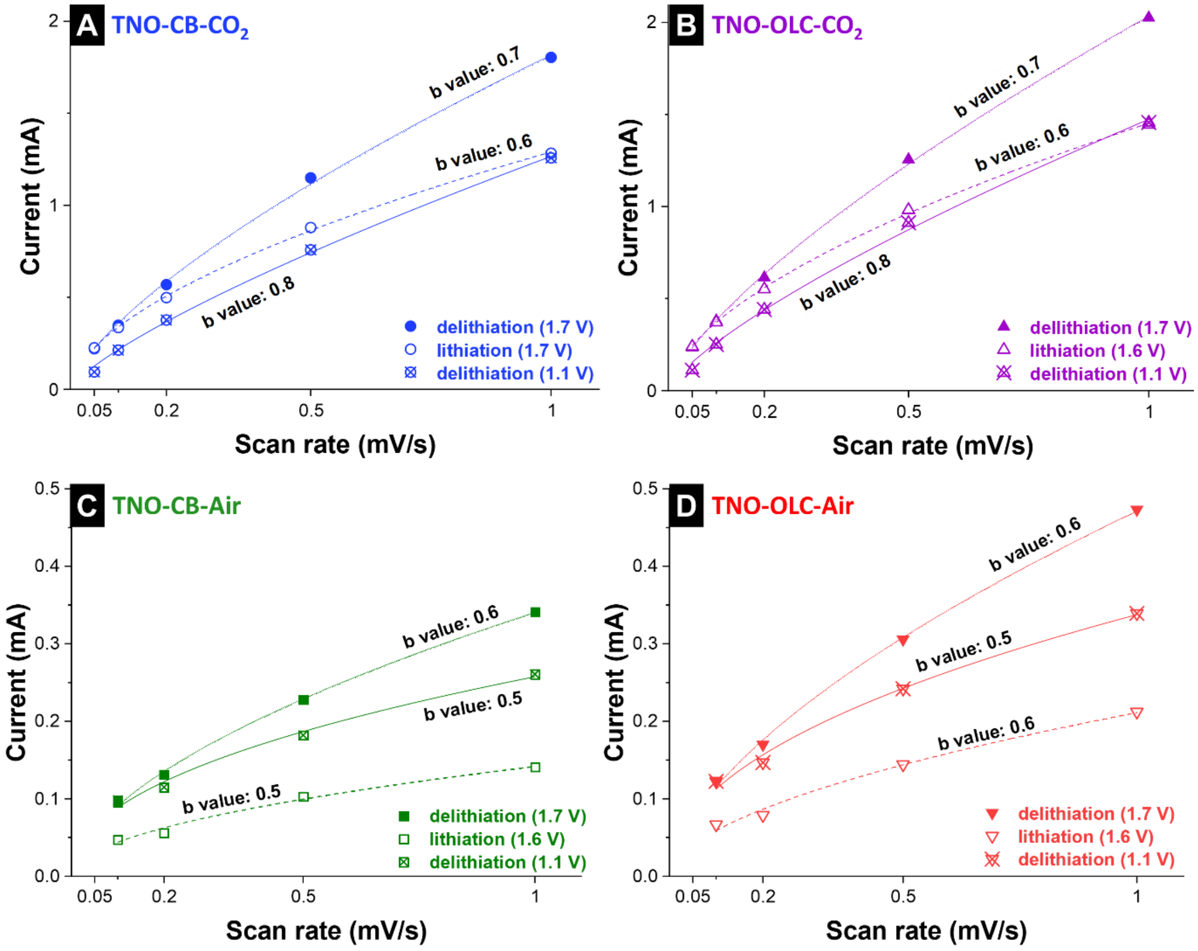


**Fig. S6.** Kinetic analysis of (A) TNO-CB-$CO_2$, (B) TNO-OLC-$CO_2$, (C) TNO-CB-Air, and (D) TNO-OLC-Air calculated from cyclic voltammograms at the different scan rates for the potential window of 0.05-2.5 V vs. $Li/Li^+$.

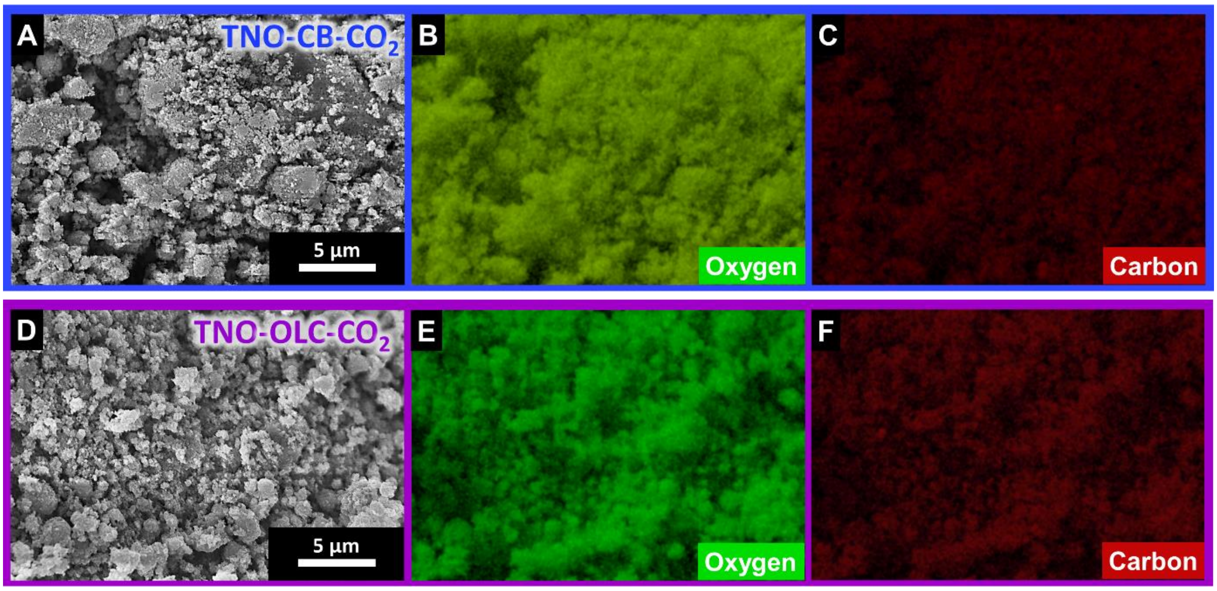


**Fig. S7.** (A-C) Analysis of the TNO-CB-$CO_2$ electrode: (A) Scanning electron micrographs, and (B-C) elemental mapping by EDX. (D-F) Analysis of the TNO-OLC-$CO_2$ electrode: (D) Scanning electron micrographs, and (E-F) elemental mapping by EDX.

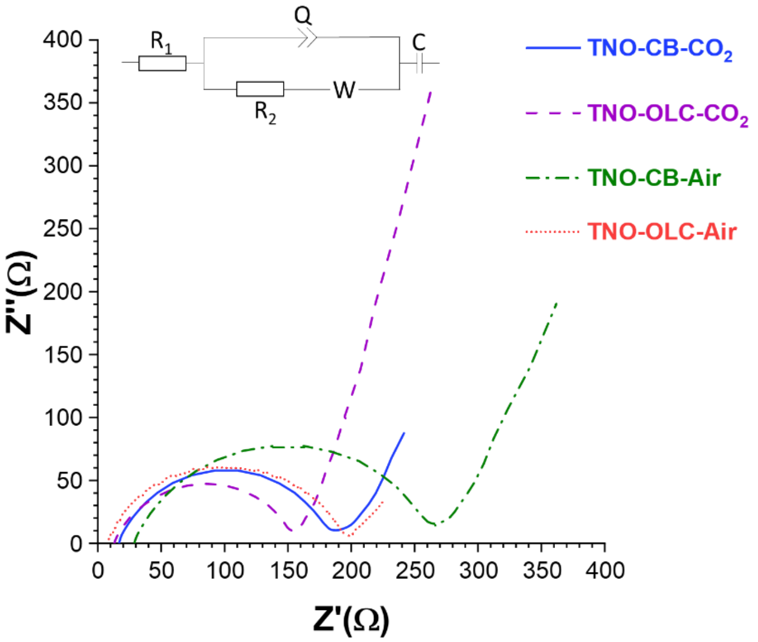


**Fig. S8.** Nyquist plot of hybrid and non-hybrid materials. The inset displays the equivalent circuit used for fitting of the electrochemical impedance spectra.

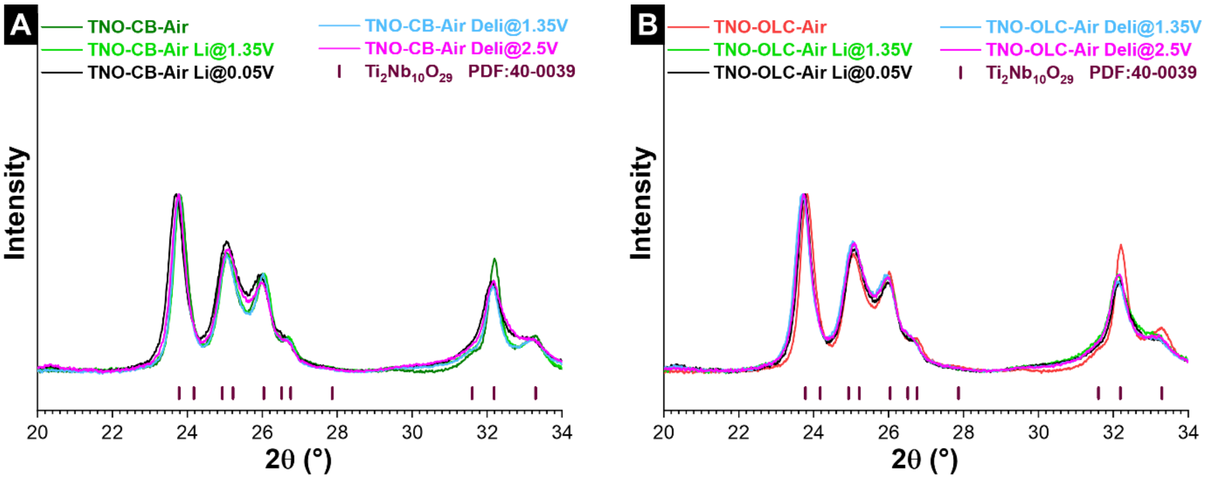


**Fig. S9.** X-ray diffraction patterns of (A) TNO-CB-Air, and (B) TNO-OLC-Air electrodes at the different lithiated/delithiated states (denoted as “Li” and “Deli”).

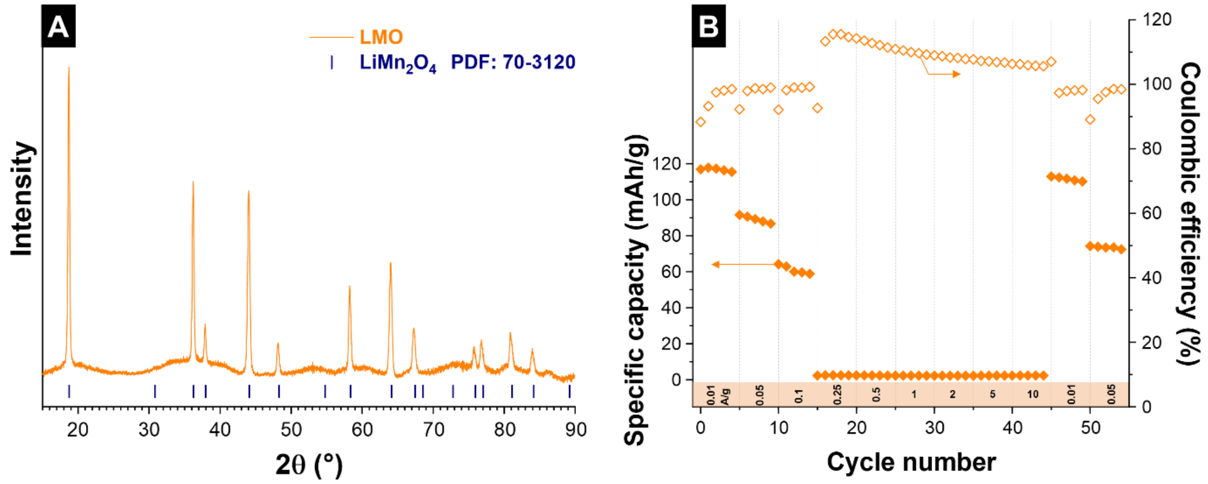


**Fig. S10.** (A) X-ray diffractogram of $LiMn_2O_4$ (LMO) with the reported Bragg positions from PDF 70-3120. (B) Rate handling performance of LMO.

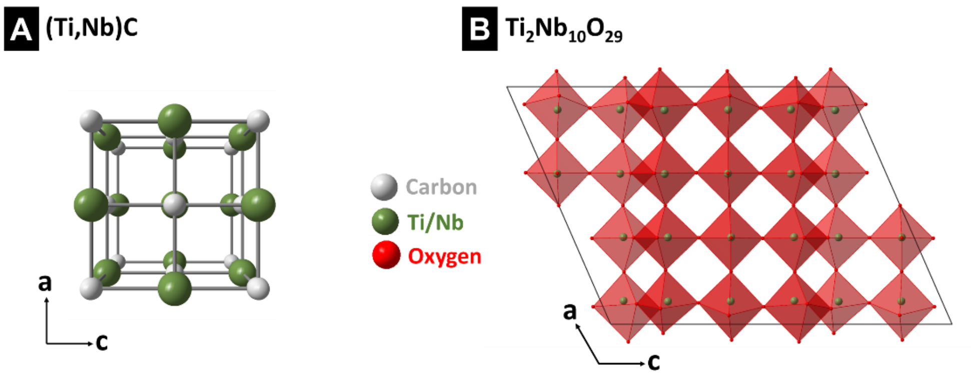


**Fig. S11.** Structural illustrations of the crystal lattice of (A) titanium niobium carbide (Ti,Nb)C, and (B) $Ti_2Nb_{10}O_{29}$.

**Table S1.** Rietveld refinement results of TNC-CB and TNC-OLC.

| Sample | Phase | Space group | Lattice parameter (Å) | Volume ($Å^3$) | Domain size (nm) | Amount (mass%) | $R_{wp}$ (%) |
|---|---|---|---|---|---|---|---|
| TNC-CB | (Ti,Nb)C | $Fm\bar{3}m$ | *a* = 4.43(4) | 87.09(4) | 19.7(4) | 98 | 2.3 |
| | WC | $P\bar{6}m2$ | *a* = 2.90(3)<br>*c* = 2.85(3) | 20.82(3) | >200 | 2 | |
| TNC-OLC | (Ti,Nb)C | $Fm\bar{3}m$ | *a* = 4.43(4) | 88.12(4) | 15.3(4) | 98 | 2.5 |
| | WC | $P\bar{6}m2$ | *a* = 2.90(6)<br>*c* = 2.85(6) | 20.76(6) | >200 | 2 | |

**Table S2.** Le Bail analysis results of the analysis of X-ray diffraction patterns of TNO-CB-$CO_2$, TNO-OLC-$CO_2$, TNO-CB-Air, and TNO-OLC-Air.

| Sample | Phase | Space group | Lattice parameter (Å) | Interaxial angle (°) | Volume ($Å^3$) | Domain size (nm) | $R_{wp}$ (%) |
|---|---|---|---|---|---|---|---|
| TNO-CB-$CO_2$ | $Ti_2Nb_{10}O_{29}$ | A12/*m*1 | *a* = 15.64<br>*b* = 3.82<br>*c* = 20.52 | β = 113.4 | 1124.1 | 55 | 3.4 |
| TNO-OLC-$CO_2$ | $Ti_2Nb_{10}O_{29}$ | A12/*m*1 | *a* = 15.62<br>*b* = 3.81<br>*c* = 20.52 | β = 113.4 | 1122.4 | 63 | 3.7 |
| TNO-CB-Air | $Ti_2Nb_{10}O_{29}$ | A12/*m*1 | *a* = 15.71<br>*b* = 3.85<br>*c* = 20.61 | β = 113.4 | 1145.3 | 56 | 3.5 |
| TNO-OLC-Air | $Ti_2Nb_{10}O_{29}$ | A12/*m*1 | *a* = 15.71<br>*b* = 3.85<br>*c* = 20.62 | β = 113.4 | 1146.0 | 88 | 3.6 |

**Table S3.** Electrochemical performance comparison of titanium niobium oxides ($TiNb_2O_7$, and $Ti_2Nb_{10}O_{29}$). CNTs: carbon nanotubes. CF: carbon fiber. rGO: reduced graphene oxide. CNFs: carbon nanofibers. CC: carbon cloth. DEC: diethylene carbonate. DMC: dimethyl carbonate. EC: ethylene carbonate. EMC: ethyl methyl carbonate. PC: propylene carbonate. MSR: Mechanically-induced self-sustaining reaction.

| Materials | Synthesis | Capacity at a low rate (mAh/g, A/g) | Capacity at a high rate (mAh/g, A/g) | Voltage range (V vs. Li/Li$^+$) | Capacity retention (%, cycle number) | Electrolyte | Reference |
|---|---|---|---|---|---|---|---|
| $TiNb_2O_7$/graphene hybrid | Solvothermal method | 300, 0.03 | 150, 1.5 | 1.0-3.0 | 67, 300 | 1 M $LiPF_6$ in EC:DMC | [1] |
| Nano-$TiNb_2O_7$/CNTs | Direct hydrolysis method | 300, 0.03 | 150, 4.5 | 0.8-3.0 | 97, 100 | 1 M $LiPF_6$ in EC:DMC:DEC | [2] |
| $TiNb_2O_7$/C nanoporous microspheres | Spray-drying method | 393, 0.1 | 120, 3.6 | 1.0-2.6 | 75, 300 | 1 M $LiPF_6$ in EC:DEC | [3] |
| CF/$TiNb_2O_7$ | Solvothermal method | 250, 0.25 | 175, 1.75 | 1.0-2.5 | 88, 1000 | 1 M $LiPF_6$ in EC:DMC | [4] |
| rGO-$TiNb_2O_7$ microsphere | Solvothermal method | 225, 0.23 | 25, 2.5 | 1.0-3.0 | 61, 500 | 1 M $LiPF_6$ in EC:EMC | [5] |
| $Ti_2Nb_{10}O_{29}$/C composite | Solid-state reaction | 296, 0.25 | 150, 4.5 | 1.0-2.5 | 87, 100 | 1 M $LiPF_6$ in EC:DMC | [6] |
| $Ti_2Nb_{10}O_{29}$/rGO | Solid-state reaction, ball-milling | 250, 0.03 | 100, 1 | 1.0-2.5 | 77, 50 | 1 M $LiPF_6$ in EC:DMC | [7] |
| $Ti_2Nb_{10}O_{29}$/carbon onion nanohybrid | Sol-gel method | 290, 0.01 | 169, 2 | 1.0-2.8 | 76, 800 | 1 M $LiPF_6$ in EC:DMC | [8] |
| $Ti_2Nb_{10}O_{29}$/carbon hybrid fiber | Electrospinning | 260, 0.025 | 180, 5 | 0.8-3.0 | 60, 500 | 1 M $LiPF_6$ in EC:DMC | [9] |
| $Ti_2Nb_{10}O_{29}$/C microsphere | Solvothermal method | 276, 0.27 | 215, 6.4 | 1.0-2.5 | 89, 200 | 1 M $LiPF_6$ in EC:EMC:DEC | [10] |
| CNFs/$Ti_2Nb_{10}O_{29}$/CC | Electrophoretic deposition, solvothermal method | 300, 0.03 | 200, 12 | 1.0-2.5 | 80, 1000 | 1 M $LiPF_6$ in EC:DEC | [11] |
| **TNO-CB-$CO_2$** | **MSR, $CO_2$ oxidation** | **272, 0.01**<br>**304, 0.01** | **157, 1**<br>**155, 1** | **1.0-2.5**<br>**0.05-2.5** | **82, 500**<br>**70, 500** | 1 M $LiPF_6$ in EC:DMC | **This work** |
| **TNO-OLC-$CO_2$** | **MSR, $CO_2$ oxidation** | **253, 0.01**<br>**350, 0.01** | **151, 1**<br>**144, 1** | **1.0-2.5**<br>**0.05-2.5** | **76, 500**<br>**67, 500** | 1 M $LiPF_6$ in EC:DMC | **This work** |

**Table S4.** Results of the $R_1$, and $R_2$ obtained by fitting the data from **Fig. S8**.

| Material | $R_1$ (Ω) | $R_2$(Ω) |
|---|---|---|
| TNO-CB-$CO_2$ | 16.2 | 168.8 |
| TNO-OLC-$CO_2$ | 12.7 | 138.7 |
| TNO-CB-Air | 28.2 | 234.4 |
| TNO-OLC-Air | 7.3 | 184.4 |

## References

[1] A. G. Ashish, P. Arunkumar, B. Babu, P. Manikandan, S. Sarang, M. M. Shaijumon, *Electrochim. Acta* **2015**, *176*, 285-292.
[2] C. Lin, L. Hu, C. Cheng, K. Sun, X. Guo, Q. Shao, J. Li, N. Wang, Z. Guo, *Electrochim. Acta* **2018**, *260*, 65-72.
[3] G. Zhu, Q. Li, Y. Zhao, R. Che, *ACS Appl. Mater. Interfaces* **2017**, *9*, 41258-41264.
[4] S. Shen, S. Deng, Y. Zhong, J. Wu, X. Wang, X. Xia, J. Tu, *Chin. Chem. Lett.* **2017**, *28*, 2219-2222.
[5] H. Noh, W. Choi, *J. Electrochem. Soc.* **2016**, *163*, A1042-A1049.
[6] G. Liu, B. Jin, R. Zhang, K. Bao, H. Xie, J. Guo, M. Wei, Q. Jiang, *Int. J. Hydrogen Energy* **2016**, *41*, 14807-14812.
[7] W. L. Wang, B.-Y. Oh, J.-Y. Park, H. Ki, J. Jang, G.-Y. Lee, H.-B. Gu, M.-H. Ham, *J. Power Sources* **2015**, *300*, 272-278.
[8] H. Shim, E. Lim, S. Fleischmann, A. Quade, A. Tolosa, V. Presser, *Sustainable Energy Fuels* **2019**, *3*, 1776-1789.
[9] A. Tolosa, S. Fleischmann, I. Grobelsek, A. Quade, E. Lim, V. Presser, *ChemSusChem* **2018**, *11*, 159-170.
[10] X. Liu, H. Wang, S. Zhang, G. Liu, H. Xie, J. Ma, *Electrochim. Acta* **2018**, *292*, 759-768.
[11] Y. Tang, S. Deng, S. Shi, L. Wu, G. Wang, G. Pan, S. Lin, X. Xia, *Electrochim. Acta* **2020**, *332*, 135433.
